# Analysing and exploiting the Mantin biases in RC4

**DOI:** 10.1007/s10623-017-0355-3

**Published:** 2017-03-28

**Authors:** Remi Bricout, Sean Murphy, Kenneth G. Paterson, Thyla van der Merwe

**Affiliations:** 1 ENS, Paris, France; 2 Royal Holloway, University of London, Egham, UK

**Keywords:** RC4, Cryptanalysis, Mantin bias, TLS, 94A60, 68P25

## Abstract

We explore the use of the Mantin biases (Mantin, Eurocrypt 2005) to recover plaintexts from RC4-encrypted traffic. We provide a more fine-grained analysis of these biases than in Mantin’s original work. We show that, in fact, the original analysis was incorrect in certain cases: the Mantin biases are sometimes non-existent, and sometimes stronger than originally predicted. We then show how to use these biases in a plaintext recovery attack. Our attack targets two unknown bytes of plaintext that are located close to sequences of known plaintext bytes, a situation that arises in practice when RC4 is used in, for example, TLS. We provide a statistical framework that enables us to make predictions about the performance of this attack and its variants. We then extend the attack using standard dynamic programming techniques to tackle the problem of recovering longer plaintexts, a setting of practical interest in recovering HTTP session cookies and user passwords that are protected by RC4 in TLS. We perform experiments showing that we can successfully recover 16-byte plaintexts with 80% success rate using $2^{31}$ ciphertexts, an improvement over previous attacks.

## Introduction

RC4 is a very widely-deployed stream cipher, but its usage in particular applications such as TLS and WPA/TKIP has recently come under heavy attack – see [1, 4, 5, 7–9], and the concurrent work to ours, [12]. The main idea of these attacks is to exploit known and newly discovered biases in RC4 keystreams to recover fixed plaintexts that are repeatedly encrypted under RC4. Such attacks can be realised against applications using RC4, including TLS and WPA/TKIP, and in particular lead to serious breaks in application layer protocols using TLS.

Mantin [6] showed that patterns of the form $AB{\mathcal {S}}AB$ occur in RC4 keystreams with higher probability than expected for a random sequence. Here *A* and *B* are byte values and ${\mathcal {S}}$ is an arbitrary byte string of some length *G*. Mantin’s main result can be stated as follows. Let $G \ge 0$ be a small integer and let $Z_r$ denote the *r*-th output byte produced by RC4. Under the assumption that the RC4 state is a random permutation at step *r*, then

1 $$\begin{aligned} \Pr \left( (Z_r,Z_{r+1}) = (Z_{r+G+2},Z_{r+G+3})\right) = 2^{-16}\left( 1+ \frac{e^{(-4-8G)/256}}{256}\right) . \end{aligned}$$

Note that for a truly random byte string $Z_r,\ldots ,Z_{r+G+3}$, the probability that $(Z_r,Z_{r+1}) = (Z_{r+G+2},Z_{r+G+3})$ is equal to $2^{-16}$. The relative bias is therefore equal to $e^{(-4-8G)/256}/256$, which is about 1 / 256 for small *G*.

Mantin’s biases are particularly attractive for use in attacks on RC4 because they are a) relatively large, b) numerous, and c) persistent in RC4 keystreams. Their presence was confirmed experimentally in [6, 10]. Indeed, they have already been exploited in attacks – see [7] and the concurrent work to ours, [12]. In the current paper, we make a systematic study of their use in attacking RC4 in the broadcast setting. Our main contributions can be summarised as follows:

1. We provide a more fine-grained analysis of the Mantin biases than in the original analysis [6], showing that in fact for certain values of *A* and *B*, the biases are non-existent, or, in some cases, stronger than predicted by (1). For example, we show that if $A=1$ or $B=1$, then the analysis in [6] fails, and so there is no reason to expect any bias for strings of the form $1B{\mathcal {S}} 1B$ or $A1 {\mathcal {S}} A1$. We also conducted large-scale experiments to confirm that our new analysis is correct. These results are important given the way in which the Mantin biases are used to attack RC4, for two reasons. Firstly, significant deviations from the expected bias behaviour would reduce the effectiveness of the attacks. Secondly, if the biases depended significantly on the values of *A*, *B* and *G*, and this dependence was well-understood, then it could be exploited in refined attacks on RC4 (this phenomenon was exploited in [8, 9] for RC4 as deployed in WPA/TKIP, though for different biases).
2. Fortunately, as we will see, the number of byte pairs (*A*, *B*) for which Mantin’s analysis is incorrect is small, and the average behaviour is still in-line with (1). This makes it profitable to develop a statistical framework for exploiting the Mantin biases in plaintext recovery attacks for the broadcast setting. We provide such a framework which directly leads to an algorithm that recovers adjacent pairs of unknown plaintext bytes, under the assumption (also used in [7, 12] and valid in practice for attacks against protocols like TLS) that the target plaintext bytes are in the neighbourhood of *known* plaintext bytes.
3. Importantly, and in contrast with [7, 12], our analysis enables us to make predictions about the numbers of ciphertexts needed to reliably recover target plaintext bytes. More precisely, our attack computes the *likelihood* of each possible target plaintext byte pair, and we are able to compute the distribution of the *rank* of the likelihood of the correct byte pair amongst the likelihoods of all possible pairs as a function of the number of ciphertexts *N* and the number of known plaintext bytes *T*. In particular, we can compute the values of (*N*, *T*) needed to ensure that the median value of the rank is 1, meaning that the correct plaintext is recovered with high probability. Our approach here is to use results from *order statistics*, a well-established field of statistical investigation that does not appear to have been applied extensively before in cryptanalysis.
4. Our framework extends smoothly to make predictions in practically interesting cases where, for example, some side information is known about the plaintexts, or where known plaintext bytes are present on either side of the unknown bytes.
5. We also extend the algorithm targeting just two unknown plaintext bytes to the situation where the target is a longer sequence of unknown plaintext bytes. This is a situation of practical interest in attacking session cookies [1] and passwords [4] that are protected by RC4 in TLS. We formally justify using as a likelihood estimate for a longer sequence of plaintext bytes the *sum* of the logs of the likelihoods of the overlapping pairs of adjacent bytes comprising that longer sequence. As a consequence of our summation formula for likelihoods, we are able to make use of standard methods from the literature, namely beam search and the list Viterbi algorithm [11], to find longer plaintext candidates having high likelihoods. The beam search algorithm is memory-efficient but does not provide any guarantees about the quality of its outputs; the list Viterbi algorithm is memory-intensive, but is guaranteed to output a list of candidates having the *L* highest likelihoods, where *L* is a parameter of the algorithm. In practical attacks involving cookies and passwords, this type of guarantee is sufficient, since large numbers of candidates can be tested for correctness.
6. We report on a range of experiments with the beam search and list Viterbi algorithms, evaluating their performance for different parameters. For example, using $L=2^{16}$ in the list Viterbi algorithm, $N=2^{31}$ ciphertexts, and 130 known plaintext bytes split either side of a 16-byte unknown plaintext, we are able to recover that 16-byte target plaintext with a success rate of about 80%. This is a significant improvement on the preferred attack of [1], which required around $2^{33}$ – $2^{34}$ ciphertexts, and is broadly comparable with the results obtained in [12].

### Further remarks on related work

AlFardan et al. [1] presented two attacks against RC4 in TLS, using single-byte biases in the first and double-byte Fluhrer–McGrew biases from [3] in the second. As in our work, their second attack uses a Viterbi algorithm (though only outputting a single plaintext candidate, so not a *list* Viterbi algorithm). Their second attack requires around $2^{34}$ ciphertexts to reliably recover a 16-byte target plaintext. Isobe et al. [5] also gave plaintext recovery attacks for RC4 using single-byte and double-byte biases, though their attacks were less effective than those of [1] and they did not explore in detail the applicability of the attacks to TLS.

Ohigashi et al. [7] were the first to use the Mantin biases in plaintext recovery attacks against RC4. They present an attack that targets a single unknown plaintext byte and that uses multiple Mantin biases (for different values of *G*). Roughly speaking, the unknown plaintext byte is aligned with the second “*B*” in patterns of the form $AB{\mathcal {S}}AB$ for varying sizes of ${\mathcal {S}}$, while the plaintext bytes in the other 3 positions are known; a count is made of the number of times in the RC4 output a string $AB{\mathcal {S}}AB$ is suggested for each unknown plaintext byte. In the analysis of [7], all biases are “weighted” in the same way, while, intuitively, the weaker the bias, the less reliable the information about plaintext bytes it provides. This overweights the known plaintext bytes that are far from the unknown, target bytes, and leads to a statistically sub-optimal attack. Their attack also recovers multiple plaintext bytes in a byte-by-byte fashion, meaning that if the attack goes wrong, then it tends to continue wrongly. This in turn means that the success rate of the attack decreases exponentially with the target plaintext length. Ohigashi et al. did not provide any rigorous analysis of their attacks, but instead simulated them to estimate their effectiveness.

In concurrent work to ours, Vanhoef and Piessens [12] conducted an extensive search for new biases in RC4 keystreams, and settled on using the Mantin biases in combination with the Fluhrer–McGrew biases to target the recovery of HTTP session cookies from TLS sessions. (They also presented an attack on WPA/TKIP that is based heavily on the single-byte bias attacks from [8, 9].) Like us, they use a likelihood-based analysis involving Mantin biases, but their analysis is only formalised for single values of *G*, and they simply take the products of likelihoods for different values of *G* without further formal statistical justification (though this procedure can be rigorously justified, as our work here shows). They also include in their product a likelihood term arising from the Fluhrer–McGrew biases. Given the *ad hoc* nature of their approach, they resort to (convincing) verification of attack performance via simulations. By contrast, we are able to provide an analytical approach which makes predictions about the distribution of the rank of our likelihood statistic for the correct plaintext bytes. Vanhoef and Piessens [12] extend their attacks to the recovery of multiple plaintext bytes using a list Viterbi algorithm, though without giving a formal justification as we do. They are able to obtain results for impressive values of *L*, the list size, in this algorithm. For example, their headline result is obtained using $L = 2^{23}$ and recovers a 16-byte plaintext with 94% success rate using $N=9 \cdot 2^{27}$ ciphertexts and roughly 256 known plaintext bytes on either side of the unknown bytes. However, it should be noted that this result applies for a restricted plaintext alphabet, which, as our analysis shows, can significantly boost the performance of attacks.

### Paper organisation

**Fig. 1:**
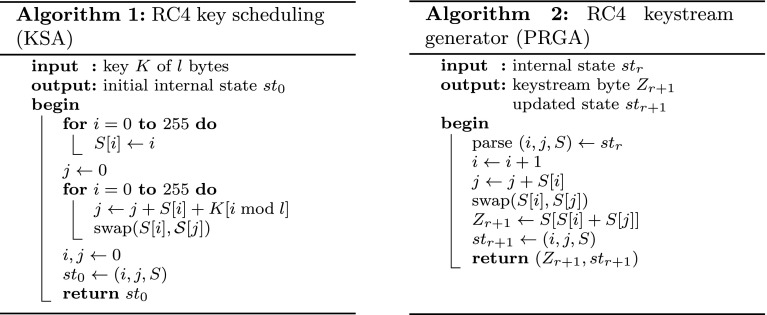
Algorithms implementing the RC4 stream cipher. All additions are performed modulo 256

In Sect. 2 we provide further background on the RC4 stream cipher. In Sect. 3, we present our refined analysis of the Mantin biases. Section 4 presents our attacks targeting adjacent pairs of unknown plaintext bytes along with their analysis using order statistics. In Sect. 5, we extend the likelihood analysis developed for pairs of unknown bytes to multiple unknown bytes, and report on our extensive experiments for this setting. Section 6 contains conclusions and open problems (Fig. 1).

## Background

### The RC4 algorithm

RC4 allows for variable-length key sizes, anywhere from 40 to 256 bits, and consists of two algorithms, namely, a *key scheduling algorithm* (KSA) and a *pseudo-random generation algorithm* (PRGA). The KSA takes as input an *l*-byte key and produces the initial internal state $st_0 = (i, j, S)$ for the PRGA; *S* is the canonical representation of a permutation of the numbers from 0 to 255 where the permutation is a function of the *l*-byte key, and *i* and *j* are indices for *S*. The KSA is specified in Algorithm 1 where *K* represents the *l*-byte key array and *S* the 256-byte state array. Given the internal state $st_r$, the PRGA will generate a keystream byte $Z_{r +1}$ as specified in Algorithm 2.

For an overview of how RC4 is used in TLS, see [1, 4]. The salient points for our analysis are as follows: in each TLS connection, RC4 is keyed with a 128-bit key that is effectively uniformly random; the key is used throughout the lifetime of a TLS connection.

### Known RC4 biases

We recall the main results on biases in RC4 outputs from [3] and [6] that are relevant here. The following is the main result of [3]:

#### Result 1

Let $Z_r$ be the r-th output byte of RC4 given a random key (of any length), where the outputs are numbered starting from 1. Then, for sufficiently large *r* and for specific values, the adjacent byte pairs $(Z_r,Z_{r+1})$ are non-uniformly distributed as shown in Table 1.

**Table 1:** Fluhrer–McGrew biases for consecutive pairs of byte values

$(Z_r,Z_{r+1})$	Condition on $i = r \bmod 256$	Probability
(0, 0)	$i=1$	$2^{-16}(1+2^{-7})$
(0, 0)	$i\ne 1, 255$	$2^{-16}(1+2^{-8})$
(0, 1)	$i\ne 0, 1$	$2^{-16}(1+2^{-8})$
$(i+1, 255)$	$i\ne 254$	$2^{-16}(1+2^{-8})$
$(255, i+1)$	$i\ne 1, 254$	$2^{-16}(1+2^{-8})$
$(255,i+2)$	$i\ne 0, 253, 254, 255$	$2^{-16}(1+2^{-8})$
(255, 0)	$i = 254$	$2^{-16}(1+2^{-8})$
(255, 1)	$i = 255$	$2^{-16}(1+2^{-8})$
(255, 2)	$i = 0, 1$	$2^{-16}(1+2^{-8})$
(129, 129)	$i = 2$	$2^{-16}(1+2^{-8})$
(255, 255)	$i \ne 254$	$2^{-16}(1-2^{-8})$
$(0, i+1)$	$i \ne 0, 255$	$2^{-16}(1-2^{-8})$

Here, *i* is the value of the internal variable of the RC4 keystream generation algorithm at the point when the first symbol of the pair is output; *i* is implemented as an 8-bit counter with wrap-around, and $i = r \bmod 256$ when the output bytes $Z_r$ of RC4 are numbered starting from 1

Extensive computations in [1] confirmed the presence of these biases and also did not reveal any other significant biases in adjacent byte pairs. Further, the biases are present from position 256 onwards.

The following result is a restatement of Theorem 1 of Mantin [6], concerning the probability of occurrence of byte strings of the form $AB{\mathcal {S}}AB$ in RC4 outputs, where *A* and *B* represent bytes and ${\mathcal {S}}$ denotes an arbitrary byte string of a particular length *G*.

#### Result 2

Let $G \ge 0$ be a small integer. Under the assumption that the RC4 state is a random permutation at step *r*, then

$$\begin{aligned} \Pr \left( (Z_r,Z_{r+1}) = (Z_{r+G+2},Z_{r+G+3})\right) = 2^{-16}\left( 1+ \frac{e^{(-4-8G)/256}}{256}\right) . \end{aligned}$$

The approximate correctness of the above result was experimentally confirmed in [6] for values of *G* up to 64 and for long keystreams. Further confirmation for the same range of *G* and for relatively short keystreams was provided in [10].

## A fine-grained analysis of the Mantin biases

The Mantin biases, as presented in Result 2, concern the probability of occurrence of byte strings of the form $AB{\mathcal {S}}AB$ in RC4 outputs. The probabilities do not depend on the specific values of *A* and *B*, but are instead averaged over these values, and depend only on the length *G* of string ${\mathcal {S}}$. Here we provide more fine-grained results about the statistics of patterns $AB{\mathcal {S}}AB$ in RC4 outputs for specific values of *A* and *B* (and in some cases, *G*). We then verify these through experiment with large numbers of RC4 outputs. All previous experimental confirmations of which we are aware only studied the dependence of the bias on *G* and so did not observe the phenomena that we catalogue below.

Our notation is the same as in [6] and in Sect. 2. Specifically, *S* denotes the RC4 permutation, and *i* and *j* are the algorithm’s internal indices. We use $S_{r}$ to denote array *S* at the end of round *r*. Similarly we use $i_{r}$ and $j_{r}$ to denote the values of *i* and *j* at the end of round *r*. Also, when studying a pattern $AB{\mathcal {S}}AB$ in the RC4 output, *G* will denote the length of the string ${\mathcal {S}}$.

### Mantin’s analysis

In [6], Mantin explains that the pattern $AB{\mathcal {S}}AB$ is more likely to arise in RC4 output than in an unbiased random byte stream because of a particular scenario that produces this type of pattern and whose probability is higher than expected. The scenario is as follows: for a given round *r*, let *g* denote $j_{r-1} - i_{r-1}$; now suppose the following three conditions are satisfied:

1. $$S_{r-1}[i_{r}] = 1$$;
2. $$j_{r+g-1} = i_{r-1}$$;
3. *i* and *j* avoid the values $i_{r-1}$, $i_{r}$, $i_{r+g-1}$ and $i_{r+g}$ from round $r+1$ to round $r+g-2$, as well as value $S_{r-1}[i_{r-1}] + S_{r-1}[j_{r-1}]$ from round *r* to round $r+g-1$, and value $S_{r}[i_{r}] + S_{r}[j_{r}]$ from round $r+1$ to round $r+g$.

Then it can be shown that the bytes output by RC4 at rounds $r+g-1$ and $r+g$ are equal to the bytes output at rounds $r-1$ and *r*, respectively. That is, a pattern $AB{\mathcal {S}}AB$ arises in the RC4 output, with ${\mathcal {S}}$ of length $G = g-2$. Mantin then goes on to evaluate the probability that these conditions hold, and, with some approximations, finally arrives at the expression in the statement of Result 2.

We now analyse this argument from [6] for special values of *A*, *B* and *g*. For each case, we will use conditions (1) and (2) to show that condition (3) cannot hold. This in turn implies that, for the special values of *A*, *B* and *g*, there is no reason to expect strings $AB{\mathcal {S}}AB$ to occur with the biased probabilities predicted by Mantin.

*Case* $A=1$: Since *A* is the output during round $r-1$, we know that

$$\begin{aligned} S_{r-1}[S_{r-1}[i_{r-1}] + S_{r-1}[j_{r-1}]] = 1. \end{aligned}$$

Moreover, because of condition (1) above, we have $S_{r-1}[i_{r}] = 1$. But $S_{r-1}$ is a permutation, which implies that $S_{r-1}[i_{r-1}] + S_{r-1}[j_{r-1}] = i_{r}$. But this is in contradiction with condition (3), since it forbids the equality $i_{k} = S_{r-1}[i_{r-1}] + S_{r-1}[j_{r-1}]$ for $r \leqslant k \leqslant r+g-1$.

*Case* $B=1$: This case is similar to the previous one. Assuming that $B=1$, we get $S_{r}[S_{r}[i_{r}] + S_{r}[j_{r}]]= 1$. Condition (1) gives $S_{r-1}[i_{r}] = 1$, so by the definition of RC4 (in particular, since it swaps *S*[*i*] and *S*[*j*] in each round), we have $S_{r}[j_{r}] = 1$. As before, $S_{r}$ is a permutation, and so its injectivity implies $S_{r}[i_{r}] + S_{r}[j_{r}] = j_{r}$. However, since $S_{r-1}[i_{r}] = 1$, we know that $j_{r} = j_{r-1} + 1$. Then, since $g=j_{r-1} - i_{r-1}$, we obtain $j_{r} = g + i_{r-1} + 1$. Finally, since *i* increments on each round, we get $j_{r} = i_{r+g}$, which provides the relation $S_{r}[i_{r}] + S_{r}[j_{r}] = i _{r+g}$, giving a contradiction with condition (3).

*Case* $A=253$ *and* $g=2$: We assume now that $A=253$ and $g=2$ (i.e. $j_{r-1} = i_{r-1} + 2$). Since $S_{r-1}[i_{r}] = 1$ (from condition (1)), we get $j_{r} = j_{r-1} +1 = i_{r-1}+3$. Condition (2) becomes $j_{r+1} = i_{r-1}$. From the behaviour of the RC4 algorithm (namely $j_{r+1} = j_{r} + S_{r}[i_{r+1}]$), we obtain $S_{r}[i_{r+1}] = 253$. Finally, since $i_{r} = i_{r+1} - 1$ and $j_{r} = i_{r+1} + 1$, the value of *S* in entry $i_{r+1}$ is not affected by round *r*, and so $S_{r-1}[i_{r+1}] = S_{r}[i_{r+1}] = 253$. On the other hand, $S_{r-1}[S_{r-1}[i_{r-1}] + S_{r-1}[j_{r-1}]] = 253$, because $A=253$. By combining these results, and noting that $S_{r-1}$ is a permutation, we get $S_{r-1}[i_{r-1}] + S_{r-1}[j_{r-1}] = i_{r+1}$ which invalidates condition (3).

*Case* $B=253$ *and* $g=2$: Because $g=2$, as in the previous case, we know that $S_{r}[i_{r+1}] = 253$. The hypothesis $B=253$ is equivalent to writing $S_{r}[S_{r}[i_{r}] + S_{r}[j_{r}]] = 253$. Then $S_{r}[i_{r}] + S_{r}[j_{r}] = i_{r+1}$, and condition (3) is contradicted again.

Note that the last two cases above concern patterns of the form *ABAB* for specific values of *A* and *B* ($G=0$), while the first two cases apply concern patterns with $A=0$ or $B=0$ for any value of $G \ge 0$. Between them, the 4 cases account for roughly 1 / 128 of all possible patterns $AB{\mathcal {S}}AB$.

### The Mantin bias when $A=B$

We now focus on refining Mantin’s estimate for biases in distributions for strings of the form $AA{\mathcal {S}}AA$ (i.e. when *A*=*B*). We will assume here that $A\ne 1$ and $B\ne 1$, since those cases were already treated above.

When $A=B$, we have that $S_{r-1}[i_{r-1}] + S_{r-1}[j_{r-1}] = S_{r}[i_{r}] + S_{r}[j_{r}]$. This is because these two values are the indices in *S* that are used for producing outputs *A* and *B* in rounds $r-1$ and *r*, respectively, and because, by assumption, the elements in these indices are not moved during these rounds. Thus Mantin’s condition (3), which states that *i* and *j* must not collide with these two values across certain rounds (amongst other things) is *more* likely to hold since the two values are equal. Specifically, the term $(1-\frac{g}{256})^2\cdot e^{-2g/256}$ in Mantin’s proof of [6, Lemma 2] can be replaced with a term $(1-\frac{g}{256})\cdot e^{-g/256}$; when $1-\frac{g}{256}$ is approximated by $e^{-g/256}$ as is the case throughout Mantin’s analysis, we finally arrive at the following:

#### Theorem 1

Let $G \ge 0$ be a small integer. Under the assumption that the RC4 state is a random permutation at step *r*, then

$$\begin{aligned} \Pr \left( (Z_r,Z_{r+1} )=(Z_{r+G+2},Z_{r+G+3}) | Z_r = Z_{r+1}\right) = 2^{-16}\left( 1+ \frac{e^{(-4-6G)/256}}{256}\right) . \end{aligned}$$

Notice here how the exponent $(-4-6G)/256$ replaces the usual exponent of $(-4-8G)/256$ appearing in Mantin’s bias, leading to *larger* biases in the special case $A=B$. Note too that this special case concerns roughly 1 / 256 of all possible patterns $AB{\mathcal {S}}AB$.

### Double-byte bias correction

As shown in Table 1, some *pairs* of bytes are more likely to occur in RC4 outputs for particular values of *i*. Some pairs are especially lucky because the bias exists for almost every value of *i*. This leads to additional biases in patterns of the form $AB{\mathcal {S}}AB$ that are not accounted for by Mantin’s analysis. In fact, the resulting biases are at least twice as big as Mantin’s for $G=0$ and do not decrease with *G*; so for $G=64$, they are ten times the size!

*Case* $A=0$ *and* $B=0$: According to Table 1, the pair of bytes (0, 0) occurs with probability $2^{-16}(1 + 2^{-8})$, instead of $2^{-16}$, for all but two values of *i*. Hence, based on the Fluhrer–McGrew biases alone, and assuming that occurrences of these biases are pair-wise independent, we would expect the pattern $00{\mathcal {S}}00$ (for any size of ${\mathcal {S}}$) to occur with probability $2^{-32}(1 + 2^{-8})^2 \approx 2^{-32}(1 + 2^{-7})$. Assuming that the generation mechanism for the Fluhrer–McGrew biases is independent of that for the Mantin biases, the occurrence probabilities can simply be summed, and we might then expect to see $00{\mathcal {S}}00$ in RC4 outputs with probability $2^{-32}\left( 1+ 2^{-8}e^{(-4-6G)/256} + 2^{-7}\right)$.

*Case* $A=0$ *and* $B=1$: Here the analysis is as in the previous case, except that, since $B=1$, we do not expect to find any Mantin bias at all. Then, for any size of ${\mathcal {S}}$, the pattern $01{\mathcal {S}}01$ can be expected to be output with probability $2^{-32}\left( 1 + 2^{-7}\right)$.

*Case* $A=255$ *and* $B=255$: In this case, Table 1 indicates that the byte pair (255, 255) occurs with probability $2^{-16}(1 - 2^{-8})$ for all but one value of *i*, that is, we have a negative bias in the majority of positions. However $A=B$, so the analysis in Sect. 3.2 applies for the Mantin bias. Following the same reasoning as before, the occurrence probability for this case is therefore expected to be $2^{-32}\left( 1+ 2^{-8}e^{(-4-6G)/256} - 2^{-7}\right)$.

Note that between them, the above 3 cases concern only a small proportion (3 out of $2^{16}$) of all possible patterns of the form $AB{\mathcal {S}}AB$.

### Experimental validation

We have conducted experiments to confirm the above theoretical observations.

We computed the distributions of patterns of the form $AB{\mathcal {S}}AB$ for values (*A*, *B*, *G*) with *A*, *B* ranging over the possible byte values and for *G* with $0 \le G \le 64$. We used $2^{38}$ RC4 keystreams with random 128-bit keys, each keystream containing $2^{12}$ bytes, for a total of $2^{50}$ keystream bytes; this computation required 72 core-days of computation on our local server (Intel Xeon cores running at 3.3Ghz, 256 GB RAM).

**Fig. 2:**
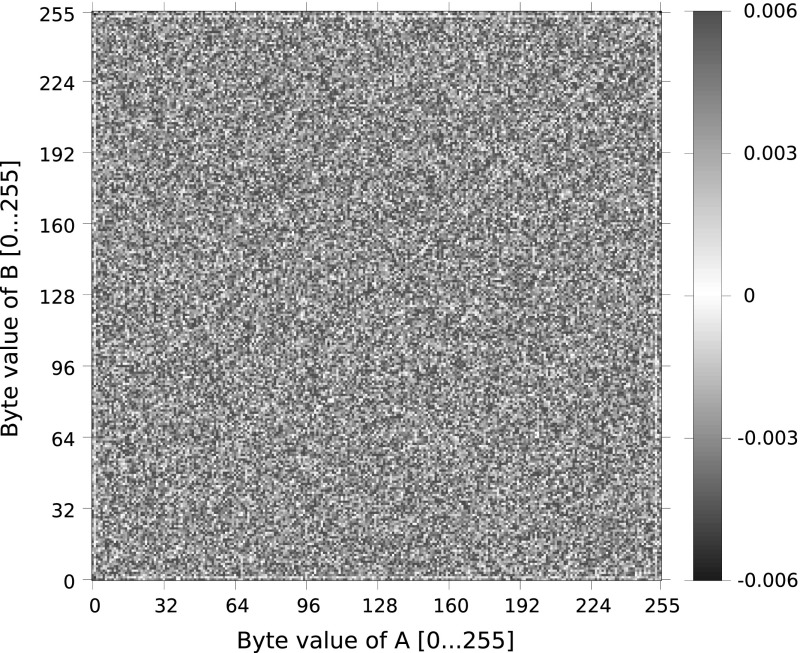
Observed biases for strings of the form *ABAB* ($G=0$) in RC4 outputs for random 128-bit keys for different values of *A* (*x*-axis) and *B* (*y*-axis). For each position we encode the bias in the keystream for the string *ABAB* as a *colour*. The colouring scheme encodes the difference between the observed probabilities and the (expected) probability $1/2^{32}$, scaled up by a factor of $2^{32}$ (Color figure online)

**Fig. 3:**
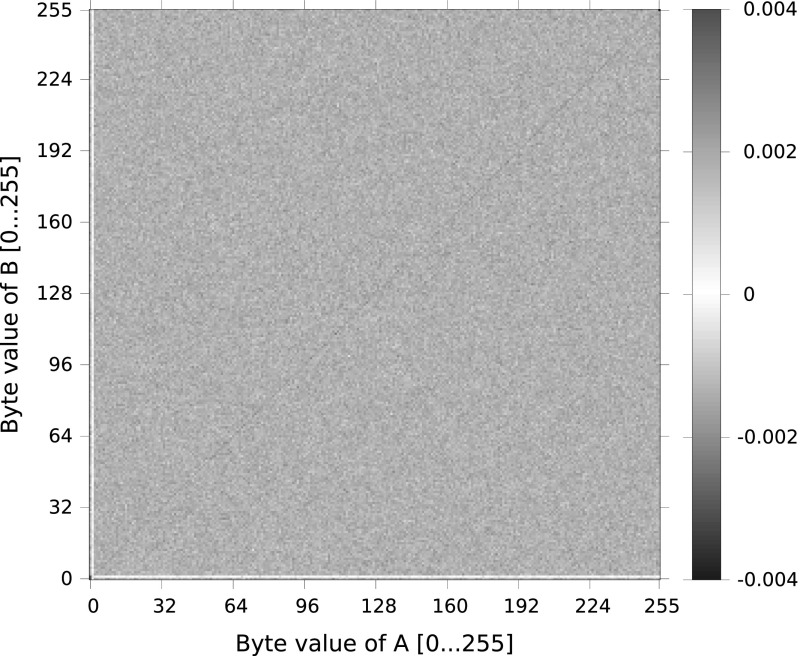
Observed biases for strings of the form $AB{\mathcal {S}}AB$ in RC4 outputs for random 128-bit keys and averaged over $0 \le G \le 64$ for different values of *A* (*x*-axis) and *B* (*y*-axis). For each position we encode the bias in the keystream for the string $AB{\mathcal {S}}AB$ as a *colour*. The colouring scheme encodes the difference between the observed probabilities and the (expected) probability $1/2^{32}$, scaled up by a factor of $2^{32}$ (Color figure online)

Our experimental results are illustrated in Figs. 2 and 3, which show the biases we observed as a function of byte values *A* and *B*, for $G=0$ ($g=2$) and aggregated over *G*, respectively. Note that these plots are predominately red, which aligns with the prediction of Mantin’s analysis that all strings $AB{\mathcal {S}}AB$ have a *positive* bias.

The data in Fig. 2 is somewhat noisy, but it is possible to see the absence of biases for $A=1$, $B=1$, $A = 253$ and $B=253$. However, when $A=B$, we do not see the positive bias behaviour predicted by Theorem 1, but instead a small, negative bias. We do not currently have an explanation for this behaviour. Coming now to Fig. 3, showing aggregated behaviour, the absence of biases for $A=1$, $B=1$ and the strong positive bias for $A=B$ are clear. It is less easy to see the deviations from Mantin’s predictions arising from the double-byte bias corrections for $(A,B) = (0,0), (0,1), (255,255)$, but they are present. Averaging over *G*, we empirically observed probabilities that were consistent with the theoretical values computed in Sect. 3.3: for $(A,B) = (0,0)$, the empirical probability was $2^{-32}(1+ 0.01005)$, for $(A,B) = (1,1)$, it was $2^{-32}(1 + 0.00834)$ and for $(A,B) = (255,255)$, it was $2^{-32}(1 - 0.00574)$.

Aside from the special case of $A=B$ and $G=0$, we did not observe any additional significant deviations from the behaviour predicted by Result 2 and our refinements of that result. However, a larger-scale computation might well reveal further fine structure. For example, as suggested by a reviewer, it is possible that there is a dependence of biases on *i*. Since *i* is known to the attacker, if such biases were present and of significant size, then this would result in exploitable behaviour.

## A plaintext recovery attack based on Mantin biases and its performance

Whilst we have observed that the distribution of patterns of the form $AB{\mathcal {S}}AB$ in RC4 outputs does not conform exactly with Mantin’s analysis [6], the deviations from the predicted behaviour are small, in the sense of affecting the probabilities of only a small proportion of the possible patterns. This means that, when the Mantin biases are used in statistical plaintext recovery attacks, it is reasonable to assume that the behaviour *is* as predicted by Result 2.

We do so henceforth, and present a plaintext recovery attack that exploits the Mantin biases. The attack is derived by first posing the plaintext recovery problem as one of maximum likelihood estimation. This enables us to also provide a concise analysis of the expected number of ciphertexts required to successfully recover the correct plaintext (and, more generally, to rank the correct plaintext within the top *R* candidates, for some chosen value of *R*).

We operate in the broadcast setting, so the same plaintext is assumed to be encrypted many times under different RC4 keystream segments, in known positions. We target the recovery of two unknown, consecutive plaintext bytes that are adjacent to a group of known plaintext bytes. These attack assumptions (partially known plaintext, broadcast setting) are fully realistic when mounting attacks that target HTTP cookies in protocols such as TLS-RC4 (see [1] for further details).

In the next section, we explain how to extend our attack targeting two consecutive plaintext bytes so as to recover longer strings of bytes.

### Maximum likelihood estimation

We consider the problem of plaintext recovery for various situations arising from RC4 encryption as a maximum likelihood problem.

#### Notational setup

The following setup applies throughout this section, unless otherwise noted. Suppose $p_1, \ldots , p_T , P_{T+1} , P_{T+2}$ are $T+2$ successive plaintext bytes which are to be encrypted a number of times under RC4 using a number of different keystreams. We suppose that the first *T* plaintext bytes $p_1 , \ldots , p_T$ are known plaintext bytes, but that the next two plaintext bytes $P_{T+1} , P_{T+2}$ are unknown and we wish to determine them. (Throughout we use lower-case letters for known quantities, and upper-case for unknown quantities, which can be regarded as random variables.)

We let $c_{i,1}, \ldots , c_{i,T} , c_{i,T+1} , c_{i,T+2}$ denote the $T+2$ successive known ciphertext bytes obtained by encrypting the plaintext bytes $p_1, \ldots , p_T , P_{T+1} , P_{T+2}$ using the $i^\mathrm{th}$ RC4 keystream $z_{i,1}, \ldots , z_{i,T} , Z_{i,T+1} , Z_{i,T+2}$. Thus we have that

$$\begin{aligned} \begin{array}{c} z_{i,1} = p_1 \oplus c_{i,1} , \> \ldots \> , z_{i,T} = p_T \oplus c_{i,T} \text{ are } \text{ known } \text{ keystream } \text{ bytes } \\ \text{ and } \\ Z_{i,T+1} = P_{T+1} \oplus c_{i,T+1} , \> , Z_{i,T+2} = P_{T+2} \oplus c_{i,T+2} \text{ are } \text{ unknown } \text{ keystream } \text{ bytes. } \\ \end{array} \end{aligned}$$

Now the Mantin bias can be expressed in the following way. We first define a positive decreasing sequence $\delta _0 , \delta _1 , \ldots , \delta _{T-2}$ by

$$\begin{aligned} \delta _G = e^{(-4 - 8 G/256)}/256 = 2^{-8} e^{- \frac{1}{64}} e^{- \frac{G}{32}} \qquad [ G = 0 ,1 , \ldots , T-2 ] . \end{aligned}$$

Then, from Result 2, we have:

$$\begin{aligned} \mathbf{P}\left( ( Z_{i,T+1} ,Z_{i,T+2} ) = ( z_{i,T-G-1} ,z_{i,T-G} ) \right) \approx 2^{-16} ( 1 + \delta _G ). \end{aligned}$$

By contrast, for byte pairs $(a_1 , a_2)$ not in the $i^\mathrm{th}$ RC4 keystream we have

$$\begin{aligned} \mathbf{P}\left( ( Z_{i,T+1} ,Z_{i,T+2} ) = ( a_1 , a_2 ) \right) \approx 2^{-16} \quad [ ( a_1 , a_2 ) \ne ( z_{i,1} , z_{i,2} ) , \ldots ( z_{i,T-1} , z_{i,T} ) ]. \end{aligned}$$

#### A likelihood function

We now calculate the probability mass function for $\theta = (P_{T+1} , P_{T+2} )$ for the $i^\mathrm{th}$ encryption based on the above probabilities. This will lead us to a likelihood function for $\theta$.

By a straightforward calculation, we have:

$$\begin{aligned} \mathbf{P}\left( (P_{T+1} , P_{T+2} ) = (p' , p'') \right) \> = \> \mathbf{P}\left( (Z_{i,T+1} , Z_{i,T+2} ) = (p' \oplus c_{i,T+1} , p'' \oplus c_{i,T+2} ) \right) . \end{aligned}$$

This probability is therefore different from $2^{-16}$ if, for some *G*, there exists a keystream byte pair $( z_{i,T-G-1} , z_{i,T-G} )$ such that

$$\begin{aligned} \begin{array}{rcl} (p' \oplus c_{i,T+1} , p'' \oplus c_{i,T+2} ) &{}=&{} (Z_{i,T+1} , Z_{i,T+2} ) = ( z_{i,T-G-1} ,z_{i,T-G} ) \\ &{}=&{} ( p_{T-G-1} \oplus c_{i,T-G-1} , p_{T-G} \oplus c_{i,T-G} ) ,\\ \end{array} \end{aligned}$$

that is to say if

$$\begin{aligned} ( p' , p'' ) = ( p_{T-G-1} \oplus c_{i,T-G-1} \oplus c_{i,T+1} , p_{T-G} \oplus c_{i,T-G} \oplus c_{i,T+2}) . \end{aligned}$$

We now let $x_{i,G}$ denote the known 2-byte quantity

$$\begin{aligned} ( p_{T-G-1} \oplus c_{i,T-G-1} \oplus c_{i,T+1} , p_{T-G} \oplus c_{i,T-G} \oplus c_{i,T+2}) \end{aligned}$$

for the $i\mathrm{th}$ RC4 encryption, and we let $x_i = ( x_{i,0} , \ldots , x_{i,T-2} )^T$ denote the vector of such known 2-byte quantities. If we then let $\theta$ denote the value of the unknown plaintext bytes $(P_{T+1} , P_{T+2} )$, then the probability mass function of $x_i$ given the parameter $\theta$ is

$$\begin{aligned} f ( x_i ; \theta ) \approx \left\{ \begin{array}{lll} 2^{-16} ( 1 + \delta _G ) &{} \phantom {++} &{} x_{i,G} = \theta \quad [G=0 , \ldots , T-2 ] \\ 2^{-16} &{}&{} \text{ otherwise. } \end{array} \right. \end{aligned}$$

This means that the likelihood function of the parameter $\theta = ( P_{T+1}, P_{T+2} )$ given the data $x_i$ is given by

$$\begin{aligned} L ( \theta ; x_i ) \approx \left\{ \begin{array}{lll} 2^{-16} ( 1 + \delta _G ) &{} \phantom {++} &{} \theta = x_{i,G} \quad [G=0 , \ldots , T-2 ] \\ 2^{-16} &{}&{} \text{ otherwise. } \end{array} \right. \end{aligned}$$

Here the approximations arise from the fact that, for a given *i*, the equality $\theta = x_{i,G}$ could hold for multiple values of *G*, while our analysis ignores this eventuality (which is of low probability).

We now consider the likelihood function of the parameter $\theta = ( P_{T+1} , P_{T+2} )$ given *N* such data vectors $x_1 , \ldots , x_N$ derived from known plaintext-ciphertext bytes. If we let

$$\begin{aligned} S_G ( \theta ; x ) = \# \{ x_{i,G} = \theta \; | \; i=1, \ldots , N \} \end{aligned}$$

be a count of the number of times the $G^\mathrm{th}$ component of $x_1 , \ldots , x_N$ is equal to $\theta$, then the joint likelihood function satisfies

$$\begin{aligned} L ( \theta ; x_1 , \ldots , x_N ) \approx 2^{-16 N} \prod _{G=0}^{T-2} ( 1 + \delta _G )^{S_G ( \theta ; x)} . \end{aligned}$$

Thus if we let *x* denote the data $x_1 , \ldots , x_N$, then the log-likelihood function is given by

$$\begin{aligned} \begin{array}{rcl} {\mathcal {L}}( \theta ; x ) = \log L ( \theta ; x ) &{}=&{}\displaystyle -16 N \log 2 + \sum _{G=0}^{T-2} S_G ( \theta ; x) \log ( 1 + \delta _G ) \\ &{}\approx &{} \displaystyle -16 N \log 2 + \sum _{G=0}^{T-2} \delta _G S_G ( \theta ;x ) \\ &{}\approx &{} \displaystyle \delta ^T S ( \theta ; x ) -16 N \log 2 , \\ \end{array} \end{aligned}$$

where $\delta = ( \delta _0 , \ldots , \delta _{T-2} )^T$ and $S ( \theta ; x ) = ( S_0 ( \theta ; x ) , \ldots , S_{T-2} ( \theta ; x ) )^T$. Thus the value of $\theta$ which maximises

$$\begin{aligned} \delta ^T S ( \theta ; x ) \approx {\mathcal {L}}( \theta ; x ) + 16 N \log 2 \end{aligned}$$

is essentially the maximum likelihood estimate $\widehat{\theta }$ of the plaintext parameter $\theta = ( P_{T+1} , P_{T+2} )$ given the known data *x*.

### Plaintext recovery attack

The preceding analysis leads immediately to an attack recovering the two unknown bytes $\theta = ( P_{T+1} , P_{T+2} )$ given access to *N* ciphertexts: for each value of $\theta$, compute $\delta ^T S ( \theta ; x )$ and output the value of $\theta$ which maximises this expression.

The attack can be implemented efficiently by processing the *i*-th ciphertext as it becomes available, using it to compute the quantities $x_{i,G}$ and updating a $(T-1) \times 2^{16}$ array of integer counters by incrementing the array in positions $(G,x_{i,G})$ for each *G* between 0 and $T-2$. Once all *N* ciphertexts are processed in this way, the array contains the counts $S_G ( \theta ; x )$ from which the log likelihood of each candidate $\theta$ can be computed by taking inner products with the vector $\delta$.

Note too that, since the attack produces log likelihood estimates for each of the $2^{16}$ candidates $\theta$, it is trivially adapted to output a ranked list of plaintext candidates in order of descending likelihood. This feature is important for our extended attacks in the following section.

This basic attack can be extended in several different ways (some of which can be considered in combination):

1. To the situation where the unknown plaintext bytes are not contiguous with the known plaintext bytes. This merely requires adjusting the above analysis to use Mantin biases for the correct values of *G* (rather than starting from $G=0$). Note that because the Mantin biases decrease in strength with increasing *G*, the attack will be rendered less effective.
2. To the case where known plaintext bytes are located on both sides of the unknown plaintext bytes (possibly in a non-contiguous fashion on one or both sides). Again, this only requires the above analysis to be adjusted to use the correct set of values for *G*. Using more biases in this way results in a stronger attack.
3. To the case where one of two target plaintext bytes, $P_{T+1}$ say, is already known. This is easily done by considering only the log likelihoods of a reduced set of candidates $\theta$ in the attack.
4. To the situation where the plaintext space is constrained in some way, for example, where the bytes of $\theta$ are known to be ASCII characters or where base64 encoding is used. Again, this can be done by working with a reduced set of candidates $\theta$.

### Distribution of the maximum likelihood statistic and attack performance

We now proceed to evaluate the effectiveness of the above basic attack, as a function of the number of available ciphertexts, *N*, and the number of known plaintext bytes, *T*.

We let $\theta ^*$ denote the true value of the plaintext parameter $\theta$. The component $S_G ( \theta ;x )$ has a binomial distribution, and there are two cases depending on whether or not $\theta$ is this true value $\theta ^*$, so we have

$$\begin{aligned} \begin{array}{rclr} S_G ( \theta ^* ;x ) &{}\sim &{} {\text{ Bin }}( N , 2^{-16} ( 1 + \delta _G ) ) \\ \text{ and } S_G ( \theta ;x ) &{}\sim &{} {\text{ Bin }}( N , 2^{-16} ) &{} \phantom {++} [ \theta \ne \theta ^* ] . \\ \end{array} \end{aligned}$$

If we write $\mu = N 2^{-16}$, then $\mathbf{E}( S_G ( \theta ^* ;x ) ) = 2^{-16} N ( 1 + \delta _G ) = \mu ( 1 + \delta _G )$ and $\mathbf{E}( S_G ( \theta ;x ) ) = 2^{-16} N = \mu$ for $\theta \ne \theta ^*$, with ${\text{ Var }}( S_G ( \theta ;x ) ) \approx 2^{-16} N = \mu$ for all $\theta$ (to a very good approximation). For the values of *N* and hence $\mu = 2^{-16} N$ of interest to us, these binomial random variables are very well-approximated by normal random variables, and we essentially have

$$\begin{aligned} \begin{array}{rclr} S_G ( \theta ^* ;x ) &{}\sim &{} {\text{ N }}( \mu ( 1 + \delta _G ) , \mu ) \\ \text{ and } S_G ( \theta ;x ) &{}\sim &{} {\text{ N }}( \mu , \mu ) &{} \phantom {++} [ \theta \ne \theta ^* ] . \\ \end{array} \end{aligned}$$

Thus the vector $S ( \theta ^* ;x ) = \left( S_0 ( \theta ^* ;x ) , \ldots , S_{T-1} ( \theta ^* ;x ) \right) ^T$ corresponding to the true parameter $\theta ^*$ and the vectors $S ( \theta ;x ) = \left( S_0 ( \theta ;x ) , \ldots , S_{T-1} ( \theta ;x ) \right) ^T$ (for $\theta \ne \theta ^*$) corresponding to other values of the plaintext parameter have a multivariate normal distribution. Furthermore, it is reasonable to assume that the components of these vectors are independent, so we have

$$\begin{aligned} \begin{array}{rclr} S ( \theta ^* ;x ) &{}\sim &{} {\text{ N }}_{T-1} ( \mu ( \mathbf{1} + \delta ) , \mu I_{T-1} ) \\ \text{ and } S ( \theta ;x ) &{}\sim &{} {\text{ N }}_{T-1} ( \mu \mathbf{1} , \mu I_{T-1} ) &{} \phantom {++} [ \theta \ne \theta ^* ] . \\ \end{array} \end{aligned}$$

The maximum likelihood statistic is essentially determined by the distributions of $\delta ^T S ( \theta ^* ;x )$ and $\delta ^T S ( \theta ;x )$ (for $\theta \ne \theta ^*$). However, these are just rank-1 linear mappings of multivariate normal random variables and so have univariate normal distributions given by

$$\begin{aligned} \begin{array}{rclr} \delta ^T S ( \theta ^* ;x ) &{}\sim &{} {\text{ N }}( \mu ( \delta ^T \mathbf{1} + | \delta |^2 ) , \mu | \delta |^2 ) \\ \text{ and } \delta ^T S ( \theta ;x ) &{}\sim &{} {\text{ N }}( \mu \delta ^T \mathbf{1} , \mu | \delta |^2 ) &{} \phantom {++} [ \theta \ne \theta ^* ] . \\ \end{array} \end{aligned}$$

The above distributions suggest that it is convenient to consider the function

$$\begin{aligned} J ( \theta ; x) = \frac{\delta ^T S( \theta ; x) - \mu \mathbf{1}^T \delta }{\mu ^{{\frac{1}{2}}} | \delta |} \ = \mu ^{- {\frac{1}{2}}} | \delta |^{-1} \left( \delta ^T S( \theta ; x) \right) - \mu ^{{\frac{1}{2}}} | \delta |^{- {\frac{1}{2}}} ( \mathbf{1}^T \delta ) \end{aligned}$$

on the parameter space. It is clear that $J ( \theta ; x)$ is a very good approximation to an affine transformation of the log-likelihood function, so the value of $\theta$ which maximises $J ( \theta ; x)$ is essentially the maximum likelihood estimate $\widehat{\theta }$ of the plaintext parameter $\theta = ( P_{T+1} , P_{T+2} )$ given the known data *x*.

We note that $J ( \theta ; x)$ has a univariate normal distribution with unit variance in both cases as we have

$$\begin{aligned} J ( \theta ^* ; x) \sim {\text{ N }}\left( \mu ^{{\frac{1}{2}}} | \delta | , 1 \right) \text{ and } J ( \theta ; x) \sim {\text{ N }}\left( 0 , 1 \right) \text{ for } \theta \ne \theta ^* . \end{aligned}$$

Furthermore, we may essentially regard all of these random variables $J ( \theta ; x )$ as independent since the random variables $S_g ( \theta ; x )$ are very close to being independent.

The function $J ( \theta ; x )$ can be thought of as a “variance-stabilised” form of log-likelihood function ${\mathcal {L}}( \theta ; x )$ of the plaintext parameter $\theta$. Furthermore, the squared length of the vector $\delta$ can be calculated as

$$\begin{aligned} | \delta |^2 = \sum _{G=0}^{T-2} \delta ^2_G = \frac{e^{\frac{1}{32}} - e^{\frac{1}{32} (3-2 T)}}{2^{16} ( e^{\frac{1}{16}} - 1 )} . \end{aligned}$$

This means, for instance, that $| \delta | \approx 0.00385$ for $T=2$ and $| \delta | \approx 0.00930$ for $T=8$, with $| \delta | \approx 0.0156$ for large *T*.

#### Performance of plaintext ranking in the basic attack

With the above reformulation, finding the maximum likelihood estimate $\widehat{ \theta }$ by maximising the function $J ( \theta ; x)$ can now be seen as essentially comparing a realisation of a normal ${\text{ N }}( \mu ^{{\frac{1}{2}}} | \delta | , 1 )$ random variable (corresponding to $J ( \theta ^* ; x )$) with a set ${\mathcal {R}} = \{ J ( \theta ; x ) | \theta \ne \theta ^* \}$ of realisations of $2^{16} - 1 = 65535$ independent standard normal ${\text{ N }}( 0 , 1 )$ random variables. Thus the maximum likelihood estimate $\widehat{\theta }$ gives the true plaintext parameter $\theta ^*$ if a realisation of an ${\text{ N }}( \mu ^{{\frac{1}{2}}} | \delta | , 1 )$ random variable exceeds the maximum of the realisations of $2^{16} - 1$ independent standard normal random variables.

This enables the probability that the maximum likelihood estimate is correct (and the basic attack succeeds) to be evaluated as a function of *N* and *T* (where, recall, *N* denotes the number of available ciphertexts and *T* denotes the number of known, consecutive plaintext bytes that are immediately followed by an unknown pair of bytes). However, we are able to go further and consider the rank of the correct plaintext $\theta ^*$ in the ordered list of values $J ( \theta ; x )$ (from highest to lowest) as a function of *N* and *T*, that is to evaluate the performance of the ranking version of the plaintext recovery attack. Such an evaluation makes use of the following result concerning order statistics [2].

##### Result 3

Suppose $X_1, \ldots , X_k$ are independent standard normal ${\text{ N }}( 0 , 1 )$ random variables and that $\varPhi$ denotes the distribution function of a standard normal ${\text{ N }}(0,1)$ random variable. Then $\varPhi ( X_1 ) , \ldots , \varPhi ( X_k )$ are independent $\text{ Uni } ( 0,1 )$ random variables and the order statistics $X_{(1)} , \ldots , X_{(k)}$ satisfy

$$\begin{aligned} \mathbf{E}\left( \varPhi ( X_{(j)} ) \right) = \frac{j}{k+1}. \end{aligned}$$

It follows that $\varPhi ( z )$ is an accurate representation on a linear uniform scale between 0 and 1 of the position of a value *z* within $X_{(1)} , \ldots , X_{(k)}$. Thus the random variable giving the position (from highest to lowest) or “rank” of $J ( \theta ^* ; x )$ within the set $\mathcal {R}$, and hence the rank of $\theta ^*$, is given accurately by rounding the random variableto the nearest integer.

The distribution function  of this (unrounded) rank  of $\theta ^*$ is given bywhere $F_{*}$ is the distribution function of $J ( \theta ^* ; x )$, that is to say of an ${\text{ N }}\left( \mu ^{{\frac{1}{2}}} | \delta | ,1 \right)$ distribution.

**Fig. 4:**
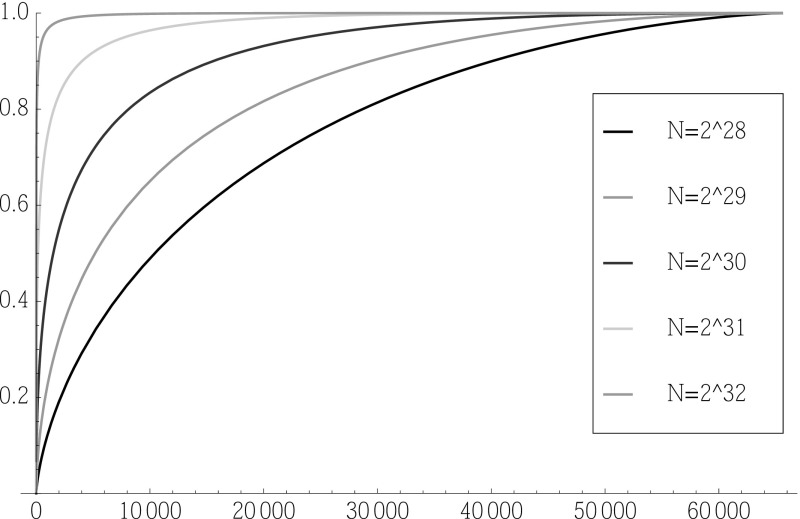
Cumulative distribution function of the rank  for different numbers of ciphertexts, *N* ($T=2^6$). The *x*-axis is a dimensionless number representing rank; the *y*-axis shows the probability that  (Color figure online)

Figure 4 shows the cumulative distribution function of the rank  for different numbers of ciphertexts, *N*, for the specific value $T=2^6$. It can be seen that as *N* approaches $2^{32}$, it becomes highly likely that the rank of $\theta ^*$ is rather small. On the other hand, when *N* drops below $2^{28}$, the attack does not have much advantage over random guessing (which would produce a diagonal line on the cumulative distribution plot).

The median of , which is very close to the mean of , is the value of *z* satisfying , that is to sayTable 2 shows some median rankings for the value of $J( \theta ^* ; x )$ within the set of all such $2^{16} = 65536$ values of $J( \theta ; x )$. A median rank of “1” indicates that the maximum likelihood estimate $\widehat{\theta }$ gives the true plaintext parameter $\theta ^*$ with high probability.

**Table 2:** Median rank of maximum likelihood estimate of plaintext parameter

*N*	$2^{27}$	$2^{28}$	$2^{29}$	$2^{30}$	$2^{31}$	$2^{32}$	$2^{33}$	$2^{34}$	$2^{35}$	$2^{36}$	$2^{37}$
$T=2^1$	28,236	26,390	23,838	20,387	15,920	10,628	5353	1596	174	3	1
$T=2^3$	22,081	18,078	13,105	7664	3024	566	25	1	1	1	1
$T=2^6$	15,735	10,423	5176	1502	155	2	1	1	1	1	1

#### Performance of plaintext ranking in variant attacks

The above analysis is easily extended to evaluate the performance of the variant attacks described in Sect. 4.2.

For variant 1, in which the unknown plaintext bytes are not contiguous with the known plaintext bytes, we need only replace the value of $|\delta |$ with the appropriate value computed from the biases actually used in the attack. For variant 2, where known plaintext bytes are located on both sides of the unknown plaintext bytes, the same is true, but this time $\delta$ increases; the analysis is otherwise identical. For example, $|\delta |^2$ doubles when we use an additional *T* known plaintext bytes $p_{T+3},\ldots ,p_{2T+2}$ in concert with $p_1,\ldots , p_{T}$. Recalling that $J ( \theta ^* ; x )$ has a ${\text{ N }}\left( \mu ^{{\frac{1}{2}}} | \delta | ,1 \right)$ distribution with $\mu = 2^{-16}N$, it can be seen that the effect of doubling $|\delta |^2$ by using “double-sided” biases in this way is the same as that of doubling *N* in the attack; put another way, using double-sided biases reduces the number of ciphertexts needed to obtain a given median ranking for the value of $J( \theta ^* ; x )$ by a factor of 2.

Variants 3 and 4 both concern the case where the plaintext space for the pair $(P_T,P_{T+1})$ is reduced from a set of $2^{16}$ candidates to some smaller set of candidates, ${\mathcal {C}}$ say. For example, in variant 3, where one of the plaintext bytes is known, $|{\mathcal {C}}|=2^8$. This means that our fundamental statistical problem becomes one of distinguishing a realisation of a normal ${\text{ N }}( \mu ^{{\frac{1}{2}}} | \delta | , 1 )$ random variable (corresponding to $J ( \theta ^* ; x )$) from a now smaller set ${\mathcal {R}} = \{ J ( \theta ; x ) | \theta \in {\mathcal {C}} \setminus \theta ^* \}$ of $|{\mathcal {C}}|-1$ realisations of independent standard normal ${\text{ N }}( 0 , 1 )$ random variables. Our previous analysis goes through as above, except that we simply replace $2^{16}$ by $|{\mathcal {C}}|$ where appropriate, resulting inThe effect of this is to divide all the entries in Table 2 by $2^{16}/|{\mathcal {C}}|$. For example, in variant 3 where $|{\mathcal {C}}|=2^8$, we would expect a median rank of roughly 6 with $N=2^{30}$ ciphertexts and $T=2^6$.

Note that these two effects are cumulative. For example, using double-sided biases and assuming one byte of plaintext from the pair $(P_{T+1},P_{T+2})$ is known has the effect of both reducing *N* by a factor of 2 *and* dividing the median rank by $2^8$. Then, for example, with only $N=2^{29}$ ciphertexts and $T=2^6$ we would expect $J ( \theta ^* ; x )$ to have a median rank of about 6, meaning that the correct plaintext $\theta ^*$ can be expected to have a high ranking.

#### Experimental validation

**Fig. 5:**
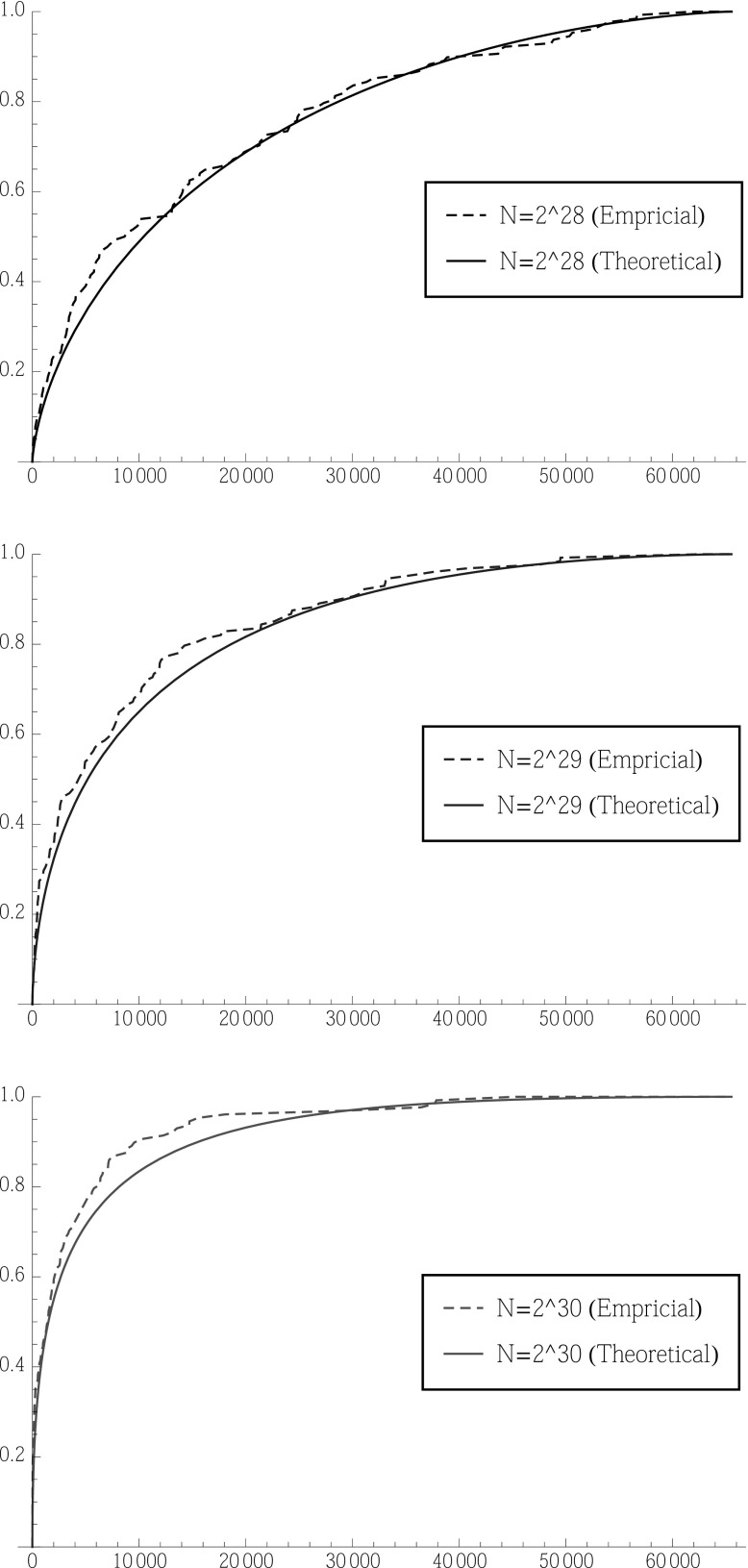
Cumulative distribution function of the rank  for different numbers of ciphertexts, *N* ($T=2^6$): $N=2^{28}$ (*top*), $N=2^{29}$ (*middle*), $N=2^{30}$ (*bottom*). In each case, the *x*-axis is a dimensionless number representing rank and the *y*-axis shows the probability that

We carried out an experimental validation of our statistical analysis, performing experiments with $T=2^6$ for different numbers of ciphertexts, *N*, and computing the cumulative distribution function of the rank . The results are shown in Fig. 5 for $N=2^{28}$, $2^{29}$ and $2^{30}$. Good agreement can be seen between the experimental results and the predictions made by our statistical analysis, with the experiments slightly outperforming the theoretical predictions in each case.

### Incorporating prior information about plaintext bytes

Prior information about the unknown plaintext bytes is frequently available and can be exploited (see, for example, [4]) to improve attacks.

Prior information in our setting can be incorporated using the inferential form of Bayes Theorem, which can be loosely expressed as

$$\begin{aligned} \text{ Posterior } \propto \text{ Likelihood } \times \text{ Prior }, \end{aligned}$$

or equivalently in its logarithmic form as

$$\begin{aligned} \text{ Log-Posterior } = \text{ Log-Likelihood } + \text{ Log-Prior } + \text{ Constant }. \end{aligned}$$

If we let $\pi ( \theta )$ denote the prior probability of the plaintext parameter $\theta = ( P_{T+1} , P_{T+2 } )$ and $\pi ( \theta ; x )$ the posterior probability of the parameter $\theta$ given the data *x*, then we have

$$\begin{aligned} \begin{array}{rcl} \log \pi ( \theta ; x ) &{}=&{} {\mathcal {L}}( \theta ; x ) + \log \pi ( \theta ) + \text{ Constant } \\ &{}\approx &{} \delta ^T S ( \theta ; x ) + \log \pi ( \theta ) + \text{ Constant }. \\ \end{array} \end{aligned}$$

This suggests that for purposes such as posterior plaintext ranking, we consider an adaptation of $J ( \theta ; x )$ given by

$$\begin{aligned} J_{\pi } ( \theta ; x) = \frac{\delta ^T S( \theta ; x) + \log \pi ( \theta ) - \mu \mathbf{1}^T \delta }{\mu ^{{\frac{1}{2}}} | \delta |} = J ( \theta ; x ) + \frac{\log \pi ( \theta )}{\mu ^{{\frac{1}{2}}} | \delta |}. \end{aligned}$$

We note that $J_{\pi } ( \theta ; x)$ has a univariate normal distribution with unit variance as we have

$$\begin{aligned} \begin{array}{crclr} &{} J_{\pi } ( \theta ^* ; x) &{}\sim &{} \displaystyle {\text{ N }}\left( \mu ^{{\frac{1}{2}}} | \delta | + \frac{\log \pi ( \theta ^* )}{\mu ^{{\frac{1}{2}}} | \delta |}, 1 \right) &{} \\ \text{ and } &{} J_{\pi } ( \theta ; x) &{}\sim &{} \displaystyle {\text{ N }}\left( \frac{\log \pi ( \theta )}{\mu ^{{\frac{1}{2}}} | \delta |} , 1 \right) &{} \text{ for } \theta \ne \theta ^* . \end{array} \end{aligned}$$

It is clear that when *N* or equivalently $\mu = 2^{-16} N$ is small, that is roughly speaking when $\mu | \delta |^2<< \left| \log \pi ( \theta ) \right|$, the mean value of the posterior scoring function is given by $\mathbf{E}\left( J_{\pi } ( \theta ; x ) \right) \approx \mu ^{- {\frac{1}{2}}} | \delta |^{-1} \log \pi ( \theta )$ for both $\theta = \theta ^*$ and $\theta \ne \theta ^*$. Thus when *N* or $\mu$ is small, the posterior scoring function essentially orders the plaintext parameters $\pi$ according to the prior distribution $\pi$; analysis of the available ciphertexts does not yield enough evidence to “overturn” the evidence given by the prior distribution. By contrast when *N* or $\mu$ is large, that is roughly speaking when $\mu | \delta |^2>> \left| \log \pi ( \theta ) \right|$, then $\mathbf{E}\left( J_{\pi } ( \theta ^* ; x ) \right) \approx \mu ^{{\frac{1}{2}}} \delta$ and $\mathbf{E}\left( J_{\pi } ( \theta ; x ) \right) \approx 0$ for $\theta \ne \theta ^*$. In this situation, the evidence of the experiment “overwhelms” the evidence given by the prior distribution, and we are essentially considering the previous scenario.

The interesting situation is therefore when $\mu | \delta |^2$ and $\left| \log \pi ( \theta ) \right|$ are of roughly comparable size. We consider how much data is needed to “overturn” an ordering of plaintext parameters according to their prior probabilities. In this situation, the scoring function for the plaintext parameter has means given by

$$\begin{aligned} \mathbf{E}\left( J_{\pi } ( \theta ^* ; x ) \right) = \mu ^{{\frac{1}{2}}} | \delta | + \frac{\log \pi ( \theta ^* )}{\mu ^{{\frac{1}{2}}} | \delta |} \quad \text{ and } \quad \mathbf{E}\left( J_{\pi } ( \theta ; x ) \right) = \frac{\log \pi ( \theta _0 )}{\mu ^{{\frac{1}{2}}} | \delta |} \> \text{ for } \theta \ne \theta ^*. \end{aligned}$$

Thus the scoring function for the correct plaintext parameter $\theta ^*$ is expected to exceed that of the plaintext parameter $\theta$ when $\mathbf{E}\left( J_{\pi } ( \theta ^* ; x ) \right) > \mathbf{E}\left( J_{\pi } ( \theta _0 ; x ) \right)$, that is to say when

$$\begin{aligned} \mu> \frac{1}{| \delta |^2} \log \frac{\pi ( \theta )}{\pi ( \theta ^* )} \quad \text{ or } \text{ equivalently } \text{ when } N > \frac{2^{16}}{| \delta |^2} \log \frac{\pi ( \theta )}{\pi ( \theta ^* )} \end{aligned}$$

The interesting case is obviously when $\pi (\theta ) > \pi ( \theta ^* )$, that is to say when $\theta$ is *a priori* a more likely plaintext parameter than $\theta ^*$. In this case, the above expression indicates how many samples are likely to be required to be able to place an *a posteriori* rank $\theta ^*$ above that for $\theta$. Clearly, the answer depends on the specifics of the distribution $\pi$.

## Attacks recovering multiple plaintext bytes

We now extend the preceding attacks and analysis to consider the situation where the target plaintext extends over multiple bytes. As in previous [1, 4, 5, 7–9] and concurrent [12] works, this is important in building practical attacks targeting HTTP cookies, passwords, etc. We are particularly interested in attack algorithms that output lists of candidates rather than single candidates, since in many practical situations, many suggested candidates can be tried one after another, as was first suggested in [1].

This problem was already addressed in [1] and [7] for attacks exploiting Fluhrer–McGrew and Mantin biases, respectively. Although not explicit in [1], the algorithm used there is a Viterbi algorithm and is guaranteed to output the best plaintext candidate on *W* bytes according to an approximate log likelihood metric; roughly $2^{33}$ – $2^{34}$ ciphertexts were needed to recover a 16-byte plaintext with high success rate. The algorithm in [7] proceeds on a byte-by-byte basis and the success probability of it recovering the correct plaintext is the product of success rates for single bytes. This, unfortunately, means that the success rate drops rapidly as a function of the byte-length of the target plaintext. For example, with $N = 2^{32}$ ciphertexts and $T=66$ known plaintext bytes, the algorithm of [7] achieves a success rate of 0.7656 for a single byte, but this would be reduced to $(0.7656)^{16} = 0.014$ for 16 bytes.

Throughout this section, we let *W* denote the byte-length of the target plaintext, and *L* the size of the list of plaintext candidates output by our plaintext recovery algorithms. An algorithm is declared successful if the target plaintext is to be found in the output list.

### A likelihood analysis for multiple plaintext bytes

As previously, we assume plaintext bytes $p_1,\ldots ,p_T$ are known. Our task now is to recover the *W* unknown bytes $\theta = (P_{T+1},\ldots ,P_{T+W})$. We let $\theta _w$ denote $(P_{T+w}, P_{T+w+1} )$ for $1 \le w \le W-1$. Using the methods of Sect. 4, we can form $W-1$ ranked lists of values for ${\mathcal {L}}( \theta _w ; x )$, where as before *x* denotes the collection of *N* data vectors $x_1 , \ldots , x_N$ derived from known plaintext-ciphertext bytes. Note here that when $w \ge 2$, these log-likelihoods will be computed using progressively weaker Mantin biases with $G \ge 1$.

To evaluate the overall log-likelihood ${\mathcal {L}}( \theta ; x )$, we will replace this quantity with the sum:

2 $$\begin{aligned} \sum _{w=1}^{W-1} {\mathcal {L}}( \theta _w ; x ) \end{aligned}$$

of log-likelihoods for the byte pairs $\theta _i$.

This replacement is formally justified as follows. Consider the probability mass function of a data vector $x_i$ given the unknown byte pairs $\theta = ( \theta _1, \ldots , \theta _{W-1} )$. This can be approximated as

$$\begin{aligned} f ( x_i ; \theta _1 , \ldots , \theta _{W-1} ) \approx \left\{ \begin{array}{lll} 2^{-16} ( 1 + \delta _{G+w-1} ) &{}&{} x_{i,G} = \theta _w \\ 2^{-16} &{}&{} \text{ otherwise. } \end{array} \right. \end{aligned}$$

Here, the nature of the approximation is similar to that made in our analysis in Sect. 4: it assumes that at most one low probability event $x_{i,G} = \theta _w$ occurs for each *i*.

However, the probability mass function of a data vector $x_i$ given a single unknown byte pair $\theta _w$ can be approximated as

$$\begin{aligned} f ( x_i ; \theta _w ) \approx \left\{ \begin{array}{lll} 2^{-16} ( 1 + \delta _{G+w-1} ) &{}&{} x_{i,G} = \theta _w \\ 2^{-16} &{}&{} \text{ otherwise, } \end{array} \right. \end{aligned}$$

so the product of all such probability mass functions can be approximated as

$$\begin{aligned} \prod _{w=1}^{W-1} f ( x_i ; \theta _w ) \approx \left\{ \begin{array}{lcl} 2^{-16(W-2)} \; 2^{-16} ( 1 + \delta _{G+w-1} ) &{}&{} x_{i,G} = \theta _w \\ 2^{-16(W-2)} \; 2^{-16} &{}&{} \text{ otherwise. } \end{array} \right. \end{aligned}$$

This enables us to give an approximate proportionality relationship between the the probability mass function of a data vector $x_i$ given the unknown byte pairs $\theta = ( \theta _1, \ldots , \theta _{W-1} )$ and the probability mass functions of a data vector $x_i$ given *single* unknown byte pairs $\theta _w$ since we now see that

$$\begin{aligned} f ( x_i ; \theta _1 , \ldots , \theta _{W-1} ) \; \propto \; \prod _{w=1}^{W-1} f ( x_i ; \theta _w ). \end{aligned}$$

This can be re-formulated in terms of likelihood functions as

$$\begin{aligned} L ( \theta ; x_i ) = L ( \theta _1 , \ldots , \theta _{W-1} ; x_i ) \; \propto \; \prod _{w=1}^{W-1} L ( \theta _w ; x_i ) . \end{aligned}$$

The likelihood function of the byte pairs $\theta = ( \theta _1 , \ldots , \theta _{W-1} )$ given all the data vectors $x = ( x_1 , \ldots , x_N )$ is therefore proportional (to a good approximation) to a product of individual likelihood functions, that is to say

$$\begin{aligned} L ( \theta ; x ) \; \propto \; \prod _{i=1}^N \left( \prod _{w=1}^{W-1} L ( \theta _w ; x_i ) \right) = \prod _{w=1}^{W-1} \left( \prod _{i=1}^N L ( \theta _w ; x_i ) \right) = \prod _{w=1}^{W-1} L ( \theta _w ; x ) , \end{aligned}$$

which can be expressed in log-likelihood terms (for some constant *C*) as

$$\begin{aligned} {\mathcal {L}}( \theta ; x ) \; \approx \; C + \sum _{w=1}^{W-1} {\mathcal {L}}( \theta _w ; x ) . \end{aligned}$$

Thus maximising the overall log-likelihood ${\mathcal {L}}( \theta ; x )$ can be achieved (to a good approximation) by maximising the sum $\sum _{w=1}^{W-1} {\mathcal {L}}( \theta _w ; x )$ of individual log-likelihoods.

### Algorithms for recovering multiple plaintext bytes

It follows from the above analysis that, to find high log-likelihood candidates for $\theta$, we need to find sequences of overlapping byte pairs $\theta _w$ for which the sums in (2) are large, given the $W-1$ lists ${\mathcal {L}}( \theta _w ; x )$. This is a classic problem in dynamic programming that can be solved by a number of different approaches. We consider two such standard approaches:

#### List Viterbi

The (parallel) list Viterbi algorithm is described in detail in [11] and generalises the usual Viterbi algorithm. In its general form it finds the *L* lowest cost state sequences through a complete trellis of some width *W* on some state space, given an initial state and a final state and where each state transition in the trellis has an associated cost. The algorithm is easily adapted to the problem at hand by setting the edge weights to be the log-likelihood values ${\mathcal {L}}( \theta _w ; x )$ and interpreting the states as byte values.^1^ Unfortunately, the algorithm is relatively memory intensive and slow, requiring roughly $256\cdot W$ times as much storage as the beam search algorithm to return a final list of *L* candidates.^2^ However, the algorithm has the advantage that it guarantees to return the *L* *best* plaintext candidates on *W* bytes, that is the top *L* candidates according to the metric represented by (2). The same algorithm appears to have been used in [12].

#### Beam search

In the beam search algorithm, we generate a list of *L* candidates on *j* positions $T+1,\ldots ,T+j$, each candidate being accompanied by a partial sum $\sum _{w=1}^{j-1} {\mathcal {L}}( \theta _w; x )$. We then expand the list to include all $256\cdot L$ candidates that are 1-byte extensions of candidates on the list, computing a new sum $\sum _{w=1}^{j} {\mathcal {L}}( \theta _w; x )$ for each candidate by adding a term ${\mathcal {L}}( \theta _w; x )$. We then prune the list back to *L* candidates again, by keeping just the top *L* candidates, but now on $w+1$ positions. The process is initialised using the top *L* values for ${\mathcal {L}}( \theta _1 ; x )$ on the first two unknown plaintext bytes. The process is finalised when $w = W-1$, and the list need not be pruned at the final step, though we do so in our implementation to provide a fair comparison with the list Viterbi algorithm. So the algorithm is deemed successful if the correct plaintext $(P_{T+1},\ldots ,P_{T+W})$ appears on the final pruned list of *L* candidates. In a further enhancement, we may assume the first and last byte of the plaintext are known, and force the candidate plaintexts to begin and end with those known bytes. The beam search algorithm is fast and memory-efficient, but does not provide any guarantees about the quality of its outputs (that is to say, we do not know if it will successfully include the highest log-likelihood plaintext on its output list).

Note that both algorithms extend smoothly to the double-sided case where some plaintext bytes are known on both sides of the *W* unknown bytes; the only modification is to the computation of the log likelihoods ${\mathcal {L}}( \theta _w ; x )$ that are input to the algorithms. Again we will be forced to use Mantin biases starting with non-zero values of *G* in computing the values ${\mathcal {L}}( \theta _w ; x )$, because of the presence of a run of unknown plaintext bytes before reaching the known plaintext bytes. Both algorithms also generalise easily to the case where the plaintext space is constrained in some way, simply by considering only restricted sets of plaintext bytes when extending candidates (in beam search) or traversing the trellis (in the list Viterbi case).

### Simulations

#### Methodology

We performed experiments with the beam search and list Viterbi algorithms, for a variety of attack parameters. We focus on recovering 16 unknown plaintext bytes, a length typical of HTTP cookies, and on attacks using single-sided and double-sided biases with, respectively, $T=66$ and 130 known plaintext bytes – in the case of List Viterbi, we require a trellis of width 18 as the first and last plaintext bytes need to be known, and for beam search we assume known plaintext bytes, one on either side of the 16 unknown target plaintext bytes. We are most interested in how the attack performance varies with *N*, the number of available ciphertexts, and *L*, the pruned list size/output list size in the two algorithms. Further experiments to explore how performance changes with *T* and *W*, and for the case of a constrained plaintext space, would be of interest, but we did not have the computing resources available to perform these. Notably, target plaintexts such as cookies often have symbols coming from a much reduced plaintext space, a fact exploited in [12] to reduce their attack’s ciphertext requirements.

Our experiments ran in two phases: in phase 1, we generated $2^{12}$ groups, each group containing $N=2^{27}$ blocks of keystream bytes. On the fly, for each group, we computed and stored the single-sided and double-sided log-likelihood measures ${\mathcal {L}}( \theta _w ; x )$ for each of the $2^{16}$ possible values of $\theta _w$ for each of 17 overlapping pairs of positions, yielding log-likelihood information for 18 consecutive unknown plaintext bytes. Then, in phase 2, we collated the measures coming from different groups to create measures for groups corresponding to progressively larger sets of blocks. This enabled us to carry out 128 plaintext recovery attacks on up to $N=2^{32}$ ciphertexts each, using our beam search and list-Viterbi algorithms. We ran each of these algorithms with $L=2^{16}$ and computed the success rate across different values of *N* (typical values of *N* are $n\cdot 2^{27}$ where $n \in \{8,10,11,12,13,14,15,16,18,20,24,28,32\}$). The properties of the list Viterbi algorithm made it easy to extract results for $L < 2^{16}$ too.

All computations were performed on the Google Compute Engine (GCE), and we optimised various parameters internal to our code for this platform. Each list Viterbi execution with $L=2^{16}$ on a trellis of width 18 took around 2 hours on a single GCE core; by contrast, the execution of the beam search algorithm completed in a only a couple of minutes for the same parameter *L*. This favourable running time inspired us to conduct further beam search experiments for higher values of *L*. For $L = 2^{17}$ each beam search experiment took about 20 minutes, and for $L = 2^{18}$, the running time was roughly 2.5 hours per experiment. We attribute this unfortunate scaling in the running time to an increasing number of cache misses as *L* grows. In total for the experiments we used around 6200 GCE core-hours of computation.

#### Results

We present our results for the attack simulations starting with those for the list Viterbi algorithm. We then discuss a number of results for the beam search algorithm and conclude this section with a comparison of the two algorithms.

**Fig. 6:**
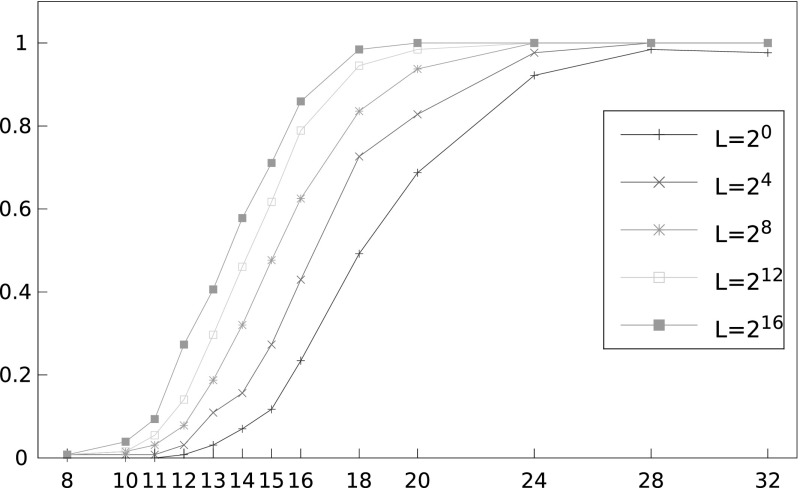
Success rate of list Viterbi algorithm in recovering a 16-byte unknown plaintext for different numbers of ciphertexts, *N* and different list sizes *L*, using double-sided biases, and 130 known plaintext bytes. The *x*-axis shows number of ciphertexts divided by $2^{27}$

*List Viterbi* Figure 6 shows how the success rate varies with *N*, the number of ciphertexts available, for the list Viterbi algorithm with double-sided biases (130 known plaintext bytes split either side of 16 unknown bytes, with 2 of the known bytes being used in the list Viterbi algorithm and the remaining 128 being used for computing log likelihoods). Each curve represents a different value of *L*. It can be seen that, for fixed *N*, the success rate increases steadily with *L* and that a threshold phenomenon is observable, where above roughly $2^{30}$ ciphertexts, the success rate takes off rapidly. For example, with $N=2^{31}$ we see a success rate 86% for $L=2^{16}$. We are confident that the success rate would continue to improve with increasing *L* and with a larger number of known plaintext bytes, bringing our results into contention with those of [12] (which used 256 known bytes instead of our 130, the significantly larger $L=2^{23}$ in the list Viterbi algorithm, and an undisclosed reduced plaintext space to achieve a success rate of 94% for recovering a 16-byte plaintext with $9\cdot 2^{27}$ ciphertexts, a little over $2^{30}$ ciphertexts).

**Fig. 7:**
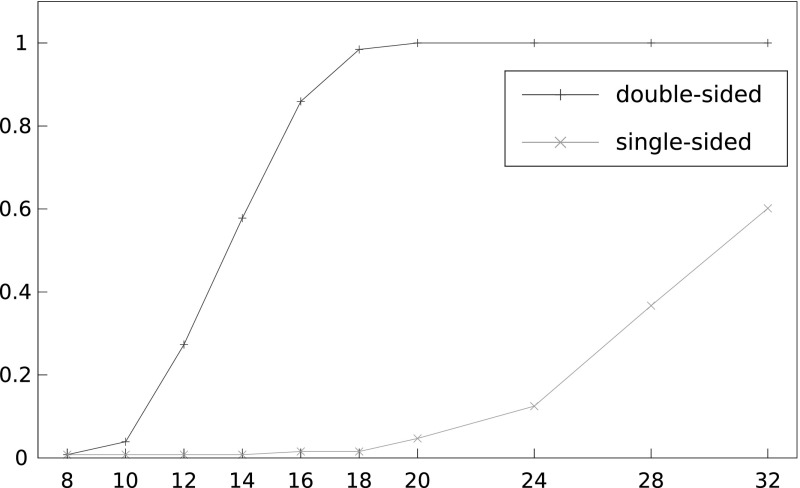
Success rate of list Viterbi algorithm in recovering a 16-byte unknown plaintext for different numbers of ciphertexts, using single-sided and double-sided biases (with 66 and 130 known plaintext bytes, respectively) and $L=2^{16}$. The *x*-axis shows number of ciphertexts divided by $2^{27}$

Figure 7 compares the performance of the single-sided and double-sided version of the attacks. Not surprisingly, the use of double-sided biases significantly improves the attack performance.

**Fig. 8:**
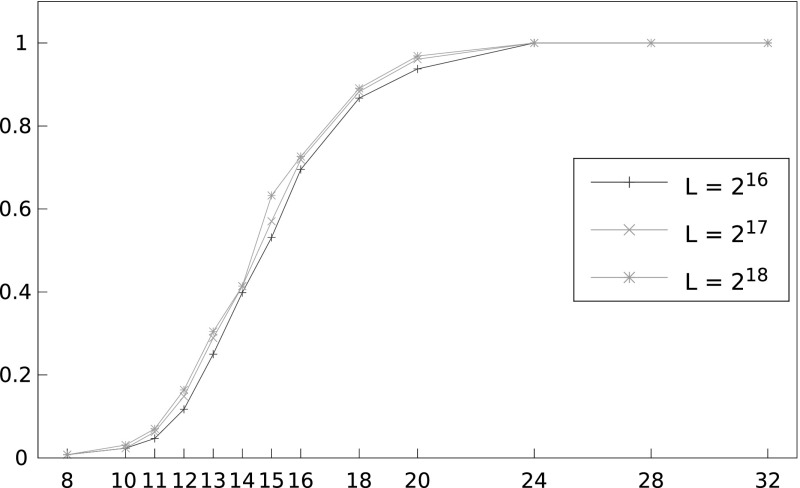
Success rate of beam search algorithm in recovering a 16-byte unknown plaintext for different numbers of ciphertexts, *N*, and different sizes of *L*, using double-sided biases and 130 known plaintext bytes. The *x*-axis shows number of ciphertexts divided by $2^{27}$

*Beam search* We note that unless otherwise stated, we use the enhancement of assuming the bytes directly adjacent to the 16 target plaintext bytes to be known, and we force our respective 18-byte candidates to start and end with these bytes. Figure 8 shows the performance of the beam search algorithm for varying numbers of ciphertexts, *N*, and for $L = 2^{16}, 2^{17}$ and $2^{18}$. As expected, we do see an improvement in success rates as *L* grows. For example, with $N = 2^{31}$ we see a success rate increase of 3% in going from $L = 2^{16}$ to $L = 2^{18}$. Significant gains, however, are likely to be made with larger values of *L*, say $L =2^{20}$.

**Fig. 9:**
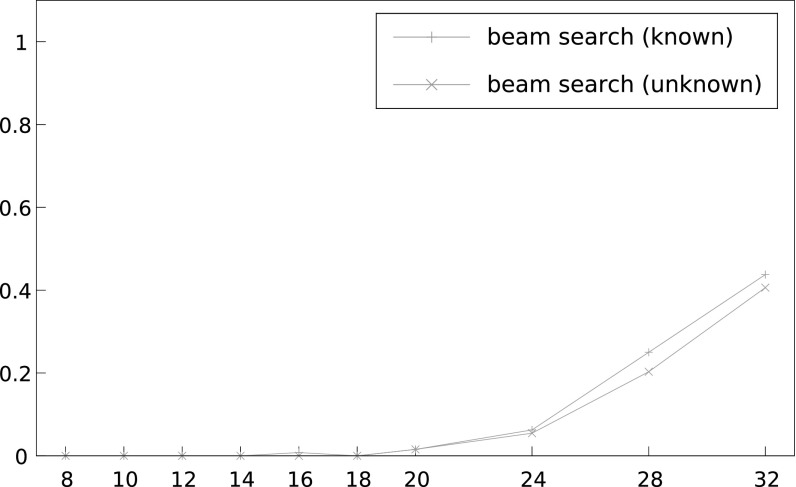
Success rate of beam search algorithm in recovering a 17-byte plaintext (first byte known) using single sided-biases with 65 known plaintext bytes compared to recovering a 16-byte unknown plaintext using single-sided biases with 64 known plaintext bytes, for different numbers of ciphertexts, *N*, and for $L = 2^{16}$. The *x*-axis shows number of ciphertexts divided by $2^{27}$

In order to determine the extent to which assuming adjacent bytes to be known improves attack performance, we ran the following two sets of experiments: We assumed the first byte adjacent to the 16 target plaintext bytes to be known and used the single-sided biases to recover 17-byte candidates (in other words, $W = 17$ with $P_{T+1}$ known). We then used the single-sided biases to recover 16 unknown target bytes ($W = 16$ and $P_{T+1}$ unknown).^3^ Figure 9 shows that there is a small advantage to using this enhancement. For instance, with $N = 2^{32}$ we see the success rate increase by 3%.

**Fig. 10:**
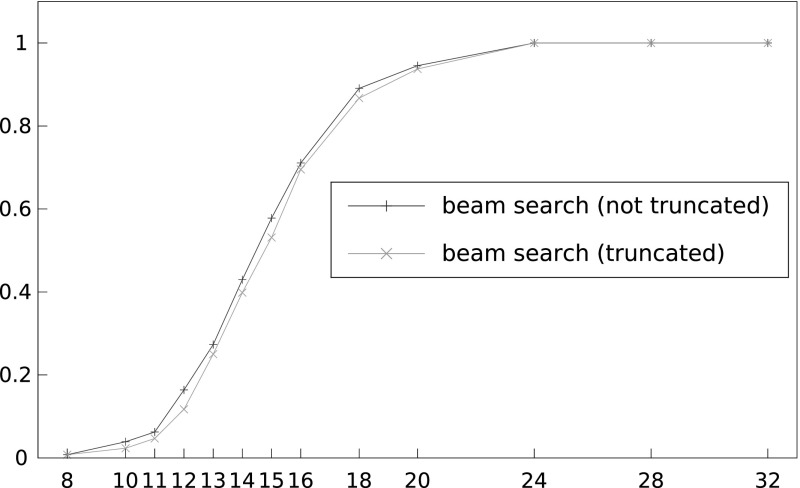
Success rate of beam search algorithm without final list pruning compared to use of final list pruning in recovering a 16-byte unknown plaintext for different numbers of ciphertexts, *N*, using double-sided biases and 130 known plaintext bytes, and for $L = 2^{16}$. The *x*-axis shows number of ciphertexts divided by $2^{27}$

In a further enhancement, we did not prune the list of plaintext candidates in the final stage of the beam search algorithm. In other words, we retained $2^8 \cdot L$ candidates in the last step of the process and declared success if the correct plaintext appeared on this larger list of candidates. Figure 10 shows the performance of the beam search algorithm using this enhancement in comparison to the case in which this enhancement is not used. We see a very slight improvement in attack performance as a result of this enhancement.

*Comparing list Viterbi and beam search*

**Fig. 11:**
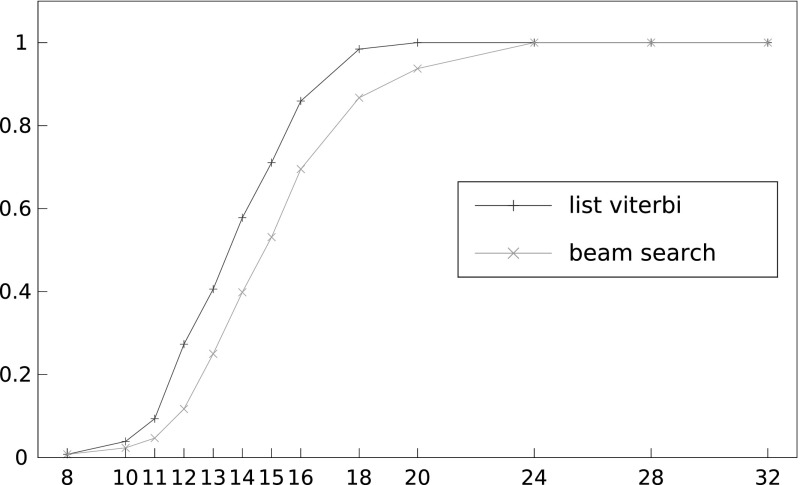
Success rate of list Viterbi algorithm compared to beam search algorithm in recovering a 16-byte unknown plaintext for different numbers of ciphertexts, *N*, using double-sided biases, $L=2^{16}$, and 130 known plaintext bytes. The *x*-axis shows number of ciphertexts divided by $2^{27}$

Figure 11 compares the performance of list Viterbi and beam search algorithms with *L* set to $2^{16}$ in both cases. It can be seen that the beam search algorithm performs very well, close to the optimal attack that is represented by list Viterbi. It may make for an attractive alternative in practice, especially for such large values of *L* where the memory consumption of the list Viterbi algorithm becomes prohibitive.

## Conclusions

In this paper, we have thoroughly analysed the Mantin biases in the outputs of the RC4 algorithm and their exploitation in plaintext recovery attacks. We showed, perhaps surprisingly, that some aspects of Mantin’s original analysis were incorrect. Our work provides an improved understanding of the genesis of the Mantin biases. We developed a statistical framework enabling us to make accurate predictions about the performance of plaintext recovery attacks targeting adjacent pairs of plaintext bytes. A particular novelty is the introduction of order statistics, enabling the expected rank of the true plaintext amongst all possible candidates to be computed. We extended the attacks to the situation of multiple unknown plaintext bytes, and provided an experimental evaluation of two different attacks for this setting, using the list Viterbi algorithm and beam search, respectively.

Several open problems are suggested by our work. It would be valuable to extend our analysis of the performance of plaintext ranking from the 2-byte setting to the multi-byte setting to yield predictive power in the latter setting, something that is currently missing from our and all other analyses. For example, it would be desirable to have a closed-form expression for the expected rank of the true plaintext candidate amongst all possible candidates as a function of the attack parameters *N*, *T*, and *W*, and of the size of the plaintext space; this would enable accurate setting of the parameter *L* (list size) when targeting a particular success rate in a real attack. It would also be interesting and useful to find a means of rigorously integrating the Fluhrer–McGrew biases and the Mantin biases in a single statistical framework, cf. the *ad hoc* approach in [12].

Finally, it would be beneficial to experiment further with our proposed multi-byte plaintext recovery algorithms. Our two-byte analysis suggests that significant gains can be expected in particular in the case of a reduced plaintext space, for example for base64 or ASCII-encoded plaintexts. These are common in session cookies and passwords, respectively. Another direction would be to integrate the use of Mantin biases with suitable plaintext language models, for example simple Markov models, in an effort to further improve the performance of plaintext recovery attacks.

## Footnotes

1 Several additional notational and conceptual changes are needed compared to the original description in [11]. In particular, the initialisation process described in [11] contains a small error, and we wish to maximise rather than minimise the cost of state sequences. The basic algorithm also requires the first and last bytes of plaintext, $P_{T+1}$ and $P_{T+W}$ to be known.

2 A low memory version of the algorithm is also given in [11] but we did not implement it.

3 Using the generated double-sided biases with $W = 18$ for the recovery of 16-byte plaintexts would have resulted in us not being able to use some of the strongest biases for plaintext recovery; targeting bytes $P_{T+2}$ to $P_{T+17}$ would mean not using biases when $G = 0$, and targeting bytes $P_{T+1}$ to $P_{T+16}$ would mean not using biases for each *G* between 0 and 2 in the recovery of $P_{T+15}$ and $P_{T+16}$.
